# Effect of caffeine supplementation on anaerobic and aerobic performance in sleep-restricted male college soccer players

**DOI:** 10.3389/fphys.2025.1561695

**Published:** 2025-03-19

**Authors:** Ruiqi Cheng, Ning Wang, Weili He, Zeng Zhou, Zhen Chen, Xiaotian Li

**Affiliations:** ^1^ Department of Physical Education, Central South University, Changsha, China; ^2^ School of Athletic Performance, Shanghai University of Sport, Shanghai, China; ^3^ School of Sports Training, Wuhan Sports University, Wuhan, China

**Keywords:** physical endurance, soccer, high-intensity exercise, psychostimulant, sleep

## Abstract

This study aimed to explore the effects of sleep restriction of ≤5 h within 24 h on anaerobic and aerobic performance in male college soccer players and evaluated the effects of acute supplementation of 3 mg⋅kg^−1^ caffeine on the aerobic and anaerobic performance of college male soccer players under sleep restriction. Methods: 10 college male soccer players were recruited, and a randomized crossover experimental design was adopted. The subjects received three intervention treatments in a randomized crossover order: Normal sleep night (NSN), sleep restriction night supplemented with placebo (SRP), and sleep restriction night supplemented with 3 mg⋅kg^−1^ caffeine (SRC), and participated in the Running-Based Anaerobic Sprint Test (RAST) and 30-15 Intermittent Fitness Test (30-15_IFT_). Results: The main effect of the RAST and the 30-15_IFT_ scores was significant (P<0.05). Post hoc analyses showed that the peak power, mean power, peak power/body mess, mean power/body mess, peak velocity mean velocity, fatigue index of the RAST, and the velocity of the intermittent fitness test (V_IFT_), maximal oxygen uptake (VO_2max_), and time to exhaustion (TTE) of the 30-15_IFT_ in the SRP group were significantly lower than those of the NSN group (P < 0.05), and the total time of the RAST was significantly higher than that of the NSN group (P < 0.05); there were significant differences between the V_IFT_, VO_2max_ and TTE indicators tested at 30-15_IFT_ between the SRC group and the SRP group (P < 0.05). Still, other indicators had no significant differences (P > 0.05). Conclusion: Sleep restriction harms the anaerobic repeated sprint and aerobic performance of college soccer players; acute supplementation of 3 mg⋅kg^−1^ of caffeine can effectively reduce the negative impact of insufficient sleep the night before on the aerobic endurance performance of college soccer players. Athletes or coaches should consider caffeine supplementation as a strategy to alleviate the negative impacts of sleep deprivation, but individual tolerance and potential side effects should be taken into account.

## 1 Introduction

Sleep is a crucial biological activity for humans, during which the body undergoes essential metabolic, immune, and cognitive processes to prepare for the next day’s physical demands (Samuels, 2008). The National Sleep Foundation recommends that healthy individuals get 7–9 h of sleep per night with 85% sleep efficiency to maintain good physiological health (Hirshkowitz et al., 2015; Xu and Li., 2024). Unfortunately, many individuals could not achieve the recommendation, especially university/college students who demonstrate even poorer patterns of sleep than other healthy adults. Many studies indicate that this cohort suffers from chronic sleep problems and disruptions (Fullagar et al., 2015). On the other hand, sleep deprivation has become increasingly prevalent among athletes, leading to issues such as inadequate sleep duration, poor sleep quality, prolonged time to fall asleep, and feelings of drowsiness and fatigue (Gupta et al., 2017; Walsh et al., 2021). Sargent et al. discovered that elite athletes believe they need about 8.3 h of sleep to properly rest their bodies, yet approximately 71% of athletes struggle to meet this requirement (Sargent et al., 2019). Previous research has highlighted the significance of sleep for both physical and mental wellbeing, showing that even just 1-2 nights of total sleep deprivation or 1–2 weeks of reduced sleep can harm cognitive performance, learning, memory, and mental health (Walsh et al., 2021). Sleep restriction (SR) occurs when individuals alter their normal sleep schedule by either staying up later or waking up earlier, leading to partial disruption of the regular sleep-wake cycle (Boonstra et al., 2007). While existing studies suggest that SR may hinder certain aspects of sports performance, the evidence is inconclusive (Fullagar et al., 2015). Mougin et al. found no effect of SR on aerobic and anaerobic performance (Mougin et al., 1991; Mougin et al., 1996), but several studies revealed that after one night of SR, power output during the Wingate test decreased (Souissi et al., 2018; Abedelmalek et al., 2013). Additionally, Abbott et al. demonstrated that SR following a soccer match did not affect Countermovement Jump (CMJ) performance the next morning (Abbott et al., 2022), while Fullager et al.'s study revealed a significant decrease in CMJ performance in the control group after experiencing SR (Fullagar H. et al., 2016). Therefore, further research is necessary to fully understand the effects of SR on various physical performance measures for athletes.

In professional soccer, it is essential to achieve a proper balance between training and game stress as well as recovery to ensure optimal physical readiness (Abbott et al., 2022), and sleep is often considered an important component of recovery (Halson, 2008). However, elite soccer players are vulnerable to sleep deprivation in a variety of ways, with Nédélec et al. noting in their review that traveling and night matches can negatively affect sleep (Nédélec et al., 2013), with 3–5 h of domestic travel disrupting sleep and increasing stress levels in elite athletes (Fowler et al., 2014). Similarly, night matches (kick-off time ≥18:00) have been shown to disrupt sleep duration and quality (Boonstra et al., 2007; Fullagar et al., 2016b; Fullagar et al., 2016a), leading to reduced physical recovery, increased stress, low mood and cognitive decline that can affect subsequent performance and increase the risk of injury (Boonstra et al., 2007). In this context, the search for effective strategies to improve sports performance under sleep restriction has become a current topic of interest in the field of competitive sports. Existing studies have mainly used various pharmacological aids to counteract the detrimental effects of sleep deprivation. Caffeine (1,3,7-trimethylxanthine) is the most commonly used central nervous system stimulant in the world, with a chemical formula of C8H10N4O2, and is found in food products such as coffee, tea, cola, and chocolate, and the use of caffeine is very common in Western countries. The limited evidence currently available suggests that caffeine has the potential to counteract the negative effects of acute sleep deprivation on sports performance. Some studies have shown that caffeine intake of 3–6 mg·kg^−1^ 1 h before exercise can effectively enhance athletic performance, significantly improving athletes' jump height, maximal strength and endurance performance with a small to moderate degree of effect (Guest et al., 2021). Additionally, the effects of caffeine on reaction time, cognitive performance and mood following sleep deprivation have been confirmed in the literature (Ace and Chaudhuri, 1987; Berzosa et al., 2011), but the evidence specifically addressing its impact under conditions of sleep restriction is limited. This gap in the literature underscores the importance of further investigation into the potential of caffeine to mitigate the negative effects of sleep deprivation, particularly in specific athletic populations such as college soccer players.

Therefore, this study aims to investigate the impact of sleep restriction on the anaerobic and aerobic performance of college male soccer players. Additionally, we will explore the effects of acute caffeine supplementation on the anaerobic and aerobic performance of college male soccer players under conditions of sleep restriction. We hypothesizes that sleep restriction negatively affects both anaerobic and aerobic performance, but caffeine supplementation may mitigate these effects.

## 2 Methods

### 2.1 Participants

15 male college soccer players were recruited for the experiment. Participants were selected based on their chronotype using the morningness/eveningness questionnaire. Only athletes with moderate and intermediate chronotypes (i.e., those who scored between 31 and 69 on the morningness/eveningness questionnaire) were recruited (Griefahn et al., 2001). Sleep diaries were collected (retiring and rising time, time in bed, sleep latency, and waking frequency and duration) for all participants during the month before the start of the experiment. Only participants with a Pittsburgh Sleep Quality Index score of less than 5 were recruited to avoid including participants with poor sleep quality (Buysse et al., 1989). Therefore, we ultimately included 10 subjects (Table 1). They were involved in approximately 3 h of training per day, 6 days a week, including strength training, and had participated in competitions for at least 4 years. All participants were free of neuromuscular or cardiorespiratory disorders, and none had a history of long-term caffeine use. The present study was conducted according to the ethical guidelines of the Declaration of Helsinki (64th World Medical Association General Assembly, Fortaleza, Brazil, October 2013). All participants signed an informed consent form and were informed of their right to withdraw at any time.

**TABLE 1:** Basic information of research participants.

Ages (yrs)	Height (cm)	Weight (kg)	Bmi (kg/m2)	Body fat rate (%)	Training years (yrs)
20.0 ± 1.3	174.6 ± 6.1	72.2 ± 8.2	23.7 ± 2.2	14.9 ± 6.1	10.2 ± 1.7

### 2.2 Experimental procedure

**FIGURE 1:**
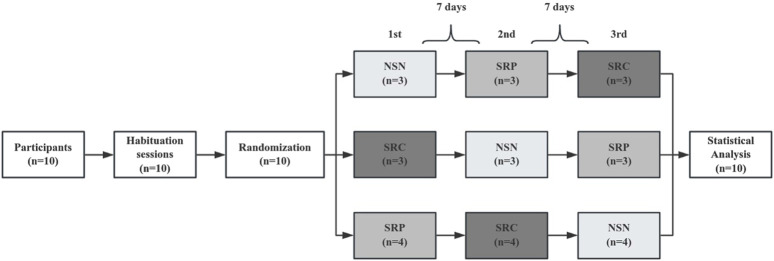
Simplified experimental protocol. NSN, normal sleep night; SRP, sleep restriction supplemented with placebo; SRC, sleep restriction supplemented with caffeine; PLA indicates xylitol placebo; CAF indicates 3 mg⋅kg^−1^ of caffeine.

Before the main experimental protocol, participants had one habituation session to familiarize themselves with the experimenter, laboratory, materials, and exercise test to minimize the learning effect and ensure exercise test reliability. In addition, each subject was given a food diary to record their food intake during the test, along with a list of caffeinated foods and beverages. Subjects were required to avoid consuming these products within 48 h prior to the test and until the end of the experimental trial. In the main experimental protocol, participants were assigned to either baseline normal sleep night (NSN), sleep restriction supplemented with a placebo (SRP), and sleep restriction supplemented with 3 mg kg^−1^ of caffeine (SRC) in random crossover order (Figure 1). There was a 1-week gap between each test day, and the entire experiment spanned over 4 weeks. All experimental sessions were initiated at 09:00 a.m. with strict termination within a fixed 2-h window to control for circadian variability. They were held in an indoor gymnasium with temperatures between 26.0°C–28.8°C and humidity ranging from 64% to 81% to reduce the impact of environmental factors. No additional verbal encouragement was given to the subjects during the tests, except to emphasize the necessary test requirements.

**FIGURE 2:**
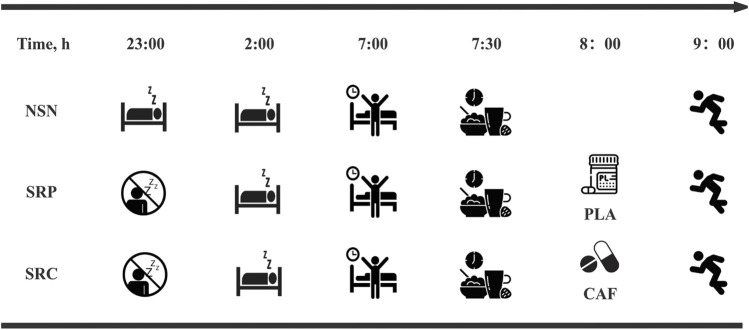
Simplified sleep intervention protocol. All times are expressed in local time (GMT+8 h). NSN, normal sleep night; SRP, sleep restriction supplemented with placebo; SRC, sleep restriction supplemented with caffeine; PLA indicates xylitol placebo; CAF indicates 3 mg⋅kg^−1^ of caffeine.

In the present study, we used an intervention of less than 5 h of sleep in a 24-hour period. The protocol designed to emulate collegiate athletes' chronic sleep profiles characterized by athletic-academic schedule conflicts (extended training + midnight study sessions) (Xu and Li, 2024). On the evenings before the SRP and SRC tests, all participants were required to participate in an online video conference from 10:00 p.m. to 2:00 a.m., and a researcher closely monitored their sleep restriction. During the sleep restriction period, the participants engaged in passive activities (e.g., reading, chatting, or playing board games) and were allowed to drink water but not eat. On the test day, the researcher awakened the participants at 7:00 a.m. They were given 30 min to overcome sleep inertia and were given a standardized breakfast (consisting of approximately 60% carbohydrate, 20% protein, and 20% fat, with a total of 5 kcal/kg) at 7:30 a.m. Within 2 hours prior to the test, the subjects' fluid intake is restricted to 5 mL/kg. There are no restrictions on the subjects' rehydration strategies during the test. During the experiment, a specific researcher administered either 3 mg kg^−1^ of caffeine (CAF) or a xylitol placebo (PLA) to the participants at 8:00 a.m. on SRP and SRC days. The participants and other researchers were unaware of which supplement was given. This was done to ensure that the double-blind method was effectively implemented. The participants took either CAF or PLA with 250 mL of warm boiled water (25°C) in red opaque gelatin capsules (identical in appearance, taste and odor). The timing of caffeine administration was determined to be 60 min before the performance test (Doyle et al., 2016). After taking the supplement, the participants rested for 55 min before undergoing the Running-Based Anaerobic Sprint Test (RAST) and 30–15 Intermittent Fitness Test (30–15_IFT_) at 9:00 a.m. in an indoor gymnasium. There was a 2-min recovery interval between the identical and different tests. The sequence of the three formal tests is shown in Figure 2, and the test order followed the guidelines outlined by the National Strength and Conditioning Association (NSCA) to minimize the impact of exercise fatigue on test performance (Haff et al., 2015).

### 2.3 Protocols

#### 2.3.1 Running-based anaerobic sprint test

**FIGURE 3:**
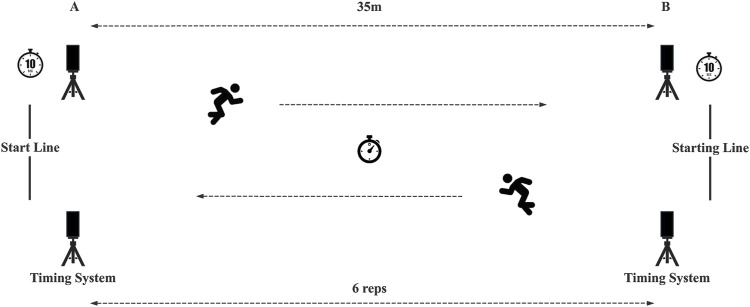
Schematic representation of the Running-Based Anaerobic Sprint Test (RAST).

The RAST is considered to be an ideal test for assessing RSA in field team athletes and consists of six short sprints of 35 m interspersed with a recovery time of 10s each (including a deceleration phase) (Zagatto et al., 2009; Keir et al., 2013) (Figure 3) At the beginning of the test, the participant is asked to sprint as fast as possible to the opposite timing gate. After a 10-s interval, the starting timing gate indicator lights up again, and the participant is asked to sprint back to the starting line. The time of each 35 m sprint was recorded and the recommended formulae were used to calculate peak power (P_peak_, W), minimum power (P_min_, W), mean power (P_mean_, W), peak velocity (V_peak_, m/s), mean velocity (V_mean_, m/s), fatigue index (FI), and total time (TT, s), and calculated peak power based on body weight (P_peak_ is defined as the highest power output recorded in the 6 sprints. P_mean_ is the average of the power calculated for all 6 sprints. The following formula was used to calculate the power output of each sprint and the fatigue index (distance, D = 35 m, time, T = measuredS sprint time):

$$P\left(W\right)=BW*\frac{D^{2}}{T^{3}}$$

$$FI=\frac{\left(P_{peak}-P_{min}\right)}{TT}$$

#### 2.3.2 30–15 intermittent fitness test

**FIGURE 4:**
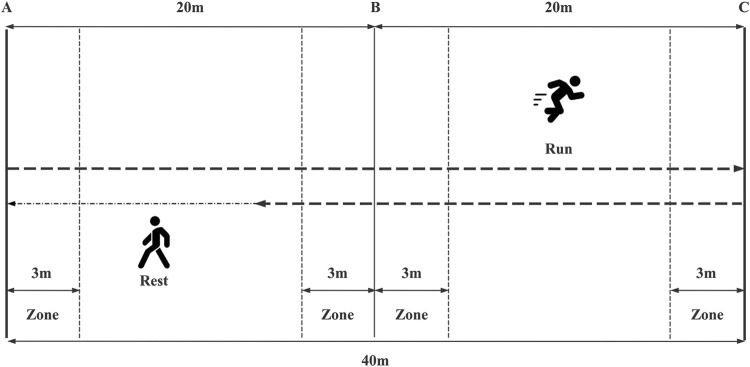
Schematic representation of 30-15 Intermittent Fitness Test (30-15_IFT_).

Aerobic performance was tested using the 30–15 _IFT_ developed by Buchheit (2010). The 30–15 _IFT_ is a traditional field test used to assess the aerobic performance of soccer players. It has demonstrated strong reliability and validity in recent research and practical applications (Valladares-Rodríguez et al., 2017; Stanković et al., 2021; Cuenca-Garcia et al., 2022). It consists of a 30-s round-trip run with a 15-s passive recovery period at each level. Participants followed a pre-recorded audio cue (APP: 30–15IFT) and started running from marker line A at 10 km/h, increasing the speed by 0.5 km/h per level. They ran back and forth between two lines 40 m apart at a certain speed during the 30 s of exercise, followed by a 15-s recovery period to walk back to within the nearest 3-m zone and the nearest marker line (A/B/C), after which the next level of testing began. Participants were verbally encouraged to perform the test as many times as they could. The test was stopped when either the participant voluntarily stopped due to exhaustion, or when they were unable to reach the next 3-m zone after the beep on three consecutive occasions. The test metrics included peak heart rate (HR_peak_, b.p.m.), mean heart rate (HR_mean_, b.p.m.), rate of perceived exertion (RPE), and the participant’s running speed (V_IFT_, m·s^-1^) and time to exhaustion (TTE) during the final completion phase, and the participant’s maximal oxygen uptake was calculated using the following formula: VO_2max_ (mL/min/kg) = 28.3–2.15G - 0.741 A- 0.0357W + 0.0586 A* V_IFT_ + 1.03 V_IFT_, wherein the above formula G stands for gender (M = 1, F = 2), A stands for age (yrs), W stands for weight (kg). Subjects' heart rates were measured throughout the 30–15_IFT_ using the Polar Team Pro System (Polar Team Pro System, Polar Electro, Kempele, Finland). Immediately after each test, we assessed their Rate of Perceived Exertion (RPE). The schematic representation of the 30–15 Intermittent Fitness Test is shown in Figure 4.

### 2.4 Statistical analysis

The data for each group were presented as mean ± standard deviation (Mean ± SD). The normality of the data was assessed using the Shapiro-Wilk test and Q-Q plots, and the homogeneity of variance was verified using the Levene test. One-way repeated measures analysis of variance (ANOVA) was performed to analyze the data for each intervention and testing session of the study. If the data did not meet the Mauchly spherical hypothesis test, the results were corrected using the Greenhouse-Geisser method. Partial eta squared (η^2^_p_) was calculated to assess the ANOVA practical significance. η^2^_p_ effect sizes were determined using the following criteria: negligible (<0.04), small (0.04–0.24), moderate (0.25–0.63), and high (>0.63) (Ferguson, 2009). When the ANOVA shows a significant main effect or interaction, we used the Bonferroni *post hoc* test and calculated Cohen’s d to quantify the magnitude of differences, with interpretation as negligible (>0.2), small (0.2–0.59), moderate (0.6–1.19), large (1.2–1.99), and very large (>2.0) (Hopkins et al., 2009). All statistical analyses were conducted using two-sided hypothesis tests, with a significance level set at α = 0.05.

## 3 Results

### 3.1 Participants

*A-priori* sample size estimation was calculated using G*Power software (Version 3.1.9.7, Düsseldorf, Germany) for an ANOVA with F-test: Repeated measures, within factors. A minimum of 9 participants were needed to detect a moderate effect size based on an expected effect size of η^2^_p_ = 0.25 (Ferguson, 2009; Smith et al., 2019; Li and Mei, 2024), assuming a correlation among repeated measures of 0.5, a significance level (α) of 0.05, and a statistical power of 0.8. Only 10 out of 15 screened volunteers completed the protocol correctly and were included in the statistical analysis. A *post hoc* analysis revealed sufficient statistical power (1-β = 0.96) at α = 0.05, exceeding the conventional threshold of 0.80.

### 3.2 RAST

**FIGURE 5:**
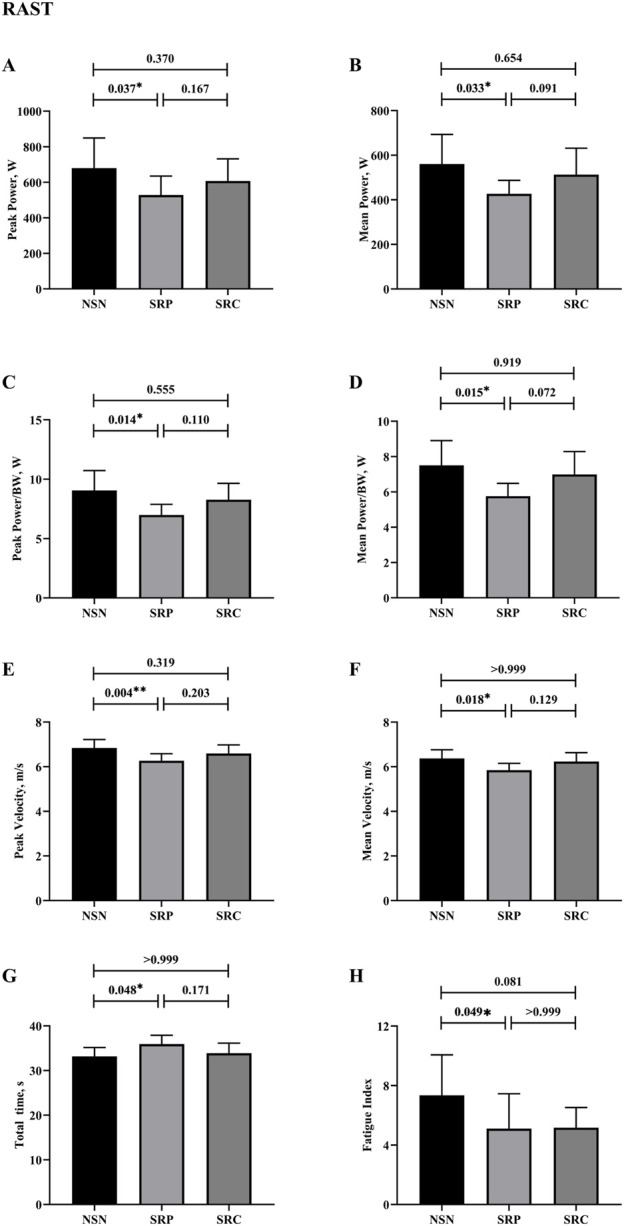
Results of running-based anaerobic sprint test. **(A)** Results of peak power. **(B)** Results of peak power/BW. **(C)** Results of mean power. **(D)** Results of mean power/BW. **(E)** Results of peak velocity. **(F)** Results of mean velocity. **(G)** Results of total time. **(H)** Results of fatigue index. * p < 0.05, **p < 0.01.

The statistical results for RAST are presented in Figure 5. There was a significant main effect on P_peak_ (F (2, 18) = 6.302, *p* = 0.008, η^2^_p_ = 0.412), P_mean_ (F (2, 18) = 6.607, *p* = 0.007, η^2^_p_ = 0.423), P_peak/BW_ (F (2, 18) = 7.421, *p* = 0.004, η^2^_p_ = 0.452) and P_mean/BW_ (F (2, 18) = 7.333, *p* = 0.005, η^2^_p_ = 0.449). Additionally, significant main effects were found for V_peak_ (F (2, 18) = 8.326, *p* = 0.004, η^2^_p_ = 0.481), V_mean_ (F (2, 18) = 6.302, *p* = 0.008, η^2^_p_ = 0.412), TT (F (2, 18) = 5.083, *p* = 0.018, η^2^_p_ = 0.361) and FI (F (2,18) = 4.966, *p* = 0.019, η^2^_p_ = 0.356). Post-hoc analysis revealed that SRP significantly reduced all performance metrics compared to the NSN. Specifically, it has a significant negative effect on P_peak_ (*p* = 0.037, d = 0.99), P_mean_, (*p* = 0.033, d = 1.01), P_peak/BW_ (*p* = 0.014, d = 1.18), P_mean/BW_ (*p* = 0.015, d = 1.17), V_peak_ (*p* = 0.004, d = 1.43), V_mean_ (*p* = 0.018, d = 1.43), TT (*p* = 0.048, d = 1.13) and FI (*p* = 0.049, d = 0.93). No significant differences were observed between SRP and SRC (*p* > 0.05). In summary, SR significantly impaired RAST performance, and caffeine supplementation did not counteract these negative effects under sleep-restricted conditions.

### 3.3 30-15_IFT_

**FIGURE 6:**
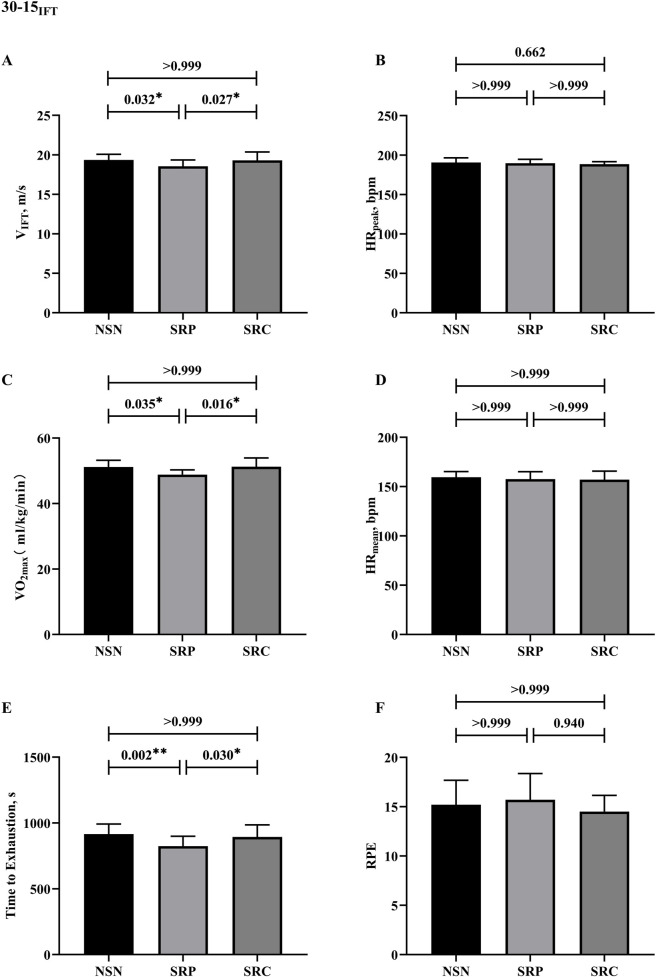
Results of 30-15 intermittent fitness test. **(A)** Results of V_IFT_. **(B)** Results of peak heart rates. **(C)** Results of VO_2max_. **(D)** Results of mean heart rates. **(E)** Results of time to exhaustion. **(F)** Results of RPE. * p < 0.05, **p < 0.01.

The results of the 30–15_IFT_ are shown in Figure 6. Significant main effects were observed for VIFT (F (2, 18) = 6.042, p = 0.010, η2p = 0.402), VO2max (F (2, 18) = 8.149, p = 0.003, η2p = 0.475), TTE (F (2, 18) = 12.498, p < 0.001, η2p = 0.581). Conversely, no significant effects were found for HRpeak (F (1.29, 18.92) = 0.645, p = 0.477, η2p = 0.067), HRmean (F (2, 18) = 0.401, p = 0.676, η2p = 0.043) and RPE (F (2, 18) = 0.401, p = 0.645, η2p = 0.067). Post-hoc comparisons indicated that SRP significantly decreased VIFT (p = 0.032, d = 1.01), VO2max (p = 0.035, d = 1.00), and TTE (p = 0.002, d = 1.55) compared to NSN. Similarly, SRC further reduced these metrics compared to SRP, with significant declines in VIFT (p = 0.027, d = −1.05), VO2max (p = 0.016, d = −1.15), and TTE (p = 0.030, d = −1.02). In conclusion, sleep restriction negatively affected 30–15IFT performance, reducing VIFT, VO2max, and TTE, whereas it had no effect on physiological metrics in 30-15IFT. In addition, caffeine supplementation significantly enhanced 30–15 IFT performance under sleep-restricted conditions.

## 4 Discussion

### 4.1 The impact of sleep restriction on performance

Sleep restriction is a condition where the normal human sleep-wake cycle is partially disturbed, leading to sleep deprivation. It can affect physical performance, as well as physiological and cognitive responses to exercise (Fullagar et al., 2015). Soccer involves numerous high-intensity intermittent activities, (e.g., repetitive sprinting, change of direction, acceleration, and deceleration) followed by a short rest period (Zhang, 2021). In the study, participants experienced significant negative changes in various RAST test metrics after ≤5 h of sleep restriction over 24 h, with the effect sizes ranging from moderate to large (0.65–1.56). P_peak_, P_mean_, P_peak/BW_, P_mean/BW_, V_peak_, and V_mean_ decreased significantly, while TT and FI increased. The decrease in performance may be due to reduced arousal and weakened motivation (Takeuchi et al., 1985; Buysse et al., 1989; Reilly and Piercy, 1994). Previous studies have shown results similar to those of the present study. Souissi et al. demonstrated a significant decrease in P_peak_ and total distance during the 5 m multiple shuttle test, as well as a significant increase in fatigue index (p < 0.001) in the Wingate test compared to the baseline night (a normal night of sleep) after a 4-h partial sleep deprivation intervention in 13 male students of physical education (Souissi et al., 2018). This suggests that partial sleep deprivation significantly affects anaerobic performance (F = 39.84, P < 0.001). Additionally, Romdhani et al. conducted the RAST on 9 highly trained male judokas under 4-h sleep deprivation conditions and showed a significant decrease in power during RAST compared to normal sleep night (P < 0.01) (Romdhani et al., 2021). Bonnet et al. suggested that sleep deprivation primarily affects the cognitive centers of the higher central nervous system. They proposed that motivation could be a key indicator related to the validity of the anaerobic performance test (Bonnet, 1980). Therefore, the decrease in performance may be due to reduced arousal and diminished motivation. In soccer matches, aerobic endurance performance is crucial for athletes. The energy supplied through the aerobic pathway accounts for approximately 90% of the total metabolic cost of a match (Bangsbo et al., 2006; Alexandre et al., 2012). Existing studies have conflicting conclusions about the impact of SR on aerobic endurance performance. Some suggest that SR does not affect single-session aerobic performance. Mejri et al. conducted a study on 10 male taekwondo players after a 3-hour sleep restriction and found no significant change in the participants' Yo-Yo IR1 (TD, HRpeak, Lac, and RPE) (p > 0.05) (Mejri et al., 2014). Mougin et al. performed 20 min of progressive incremental exercise on a cycle ergometer the following day after a 3-h sleep restriction intervention in seven male cyclists working at 75% VO_2max_ and found no significant difference in maximal exercise intensity compared to the baseline group (Mougin et al., 1991). However, our study yielded opposite results. Compared with the NSN, the participants under SR showed a significant decrease in VO_2max_, V_IFT_, and TTE. In contrast, the two groups had no differences in RPE levels at exercise termination. Although the RPE at task completion was the same, the significant reduction in TTE and V_IFT_ suggests an increase in subjective fatigue at the same levels as the 30–15_IFT_ task. Perceived exertion reflects the effort required to overcome fatigue, and according to the psychobiological model of exercise tolerance, athletes terminate task engagement when perceived effort surpasses both volitional capacity (maximum effort they are willing to exert) and perceived capability thresholds, with downstream decrements manifesting in TTE and V_IFT_ (Roberts et al., 2019). Meanwhile, given the linear nature of the 30–15_IFT_ test protocol, the reduction in V_IFT_ following sleep restriction can explain the decrease in VO_2max_. Sleep restriction impairs mood and mental alertness, and although we did not directly measure mental fatigue, we hypothesize that acute sleep restriction tasks may affect the level of mental fatigue experienced or tolerated during endurance tasks, thereby impacting endurance performance. In addition, many studies have simultaneously measured the changes in other physiological and psychological aspects of athletes during aerobic performance testing to clarify the reasons for the negative impact of sleep restriction on aerobic performance (Mougin et al., 1991; Oliver et al., 2009; Mejri et al., 2014). The existing research indicates that sleep restriction did not affect HR_peak_ and HR_mean_, which aligns with the results of our study. This implies that getting less than 5 h of sleep may have a negligible impact on cardiorespiratory fitness while engaging in aerobic exercise. In our study, despite a decrease in TTE and V_IFT_ in the SR condition compared to the NSN group, HR_peak_ and HR_mean_ remained similar. Previous studies also support the idea that physiological responses generally stay consistent during aerobic endurance exercise after sleep restriction. It is suggested that the decline in endurance performance may be due to changes in perception, such as an increase in subjective exertion, rather than impairment of the aerobic pathway (Fullagar et al., 2015). The observed increase in subjective exertion along with reduced power output may indicate a connection between the decreased central nervous drive and the neural theory of sleep.

### 4.2 The impact of caffeine

The sleep-wake cycle is crucial for human circadian rhythms, and its disruption can significantly affect behavior, as well as physical and mental performance in various situations (Souissi et al., 2015). While athletes and coaches understand the importance of sufficient sleep for athletic performance, athletes often experience disruptions in their sleep patterns due to factors such as jet lag, travel, pre-competition anxiety, and other reasons (Abedelmalek et al., 2013). Efforts to mitigate the negative effects of sleep deprivation on short-term sports performance are a significant focus in sports science. Caffeine, the world’s most commonly used stimulant, is noted by the International Society of Sports Nutrition to rapidly enhance sports performance when supplemented at 3–6 mg kg^−1^ (Guest et al., 2021). This improvement encompasses jumping, sprinting, endurance, and various sport-specific aerobic and anaerobic maneuvers. Furthermore, caffeine has been found to enhance cognitive functions, including attention and alertness. The present study indicated that acute caffeine supplementation effectively mitigated the impact of sleep deprivation on aerobic endurance exercise performance. Participants demonstrated significantly improved V_IFT_, TTE, and VO_2max_ during the 30–15_IFT_ when taking caffeine compared to a placebo (*p* < 0.05). However, caffeine did not affect RAST performance or physiological indices of 30-15_IFT_ (*p* > 0.05). A study by Kcharem et al. involving 12 runners discovered that repeated ingestion of a 6 mg/kg dose of caffeine on a night of complete sleep deprivation led to significantly improved aerobic endurance performance compared to the ingestion of a placebo (↑8.9%, P < 0.001) (Khcharem et al., 2022). It is believed that caffeine’s impact on aerobic endurance performance during sleep restriction is due to its stimulation of the central nervous system by blocking adenosine receptors (A1 and A2a). These receptors are functional isomers with dopamine receptors (D1 and D2) in different parts of the brain, leading to increased dopamine levels in the body (Ferré, 2016). Caffeine also enhances blood flow to the muscles and heart, reduces perceived exertion and pain, delays fatigue, and improves aerobic endurance performance (Doherty and Smith, 2005). It is worth noting that while caffeine can have a significant prokinetic effect, it did not affect RAST performance in this study, which is consistent with previous research. Moore et al. discovered that acute supplementation with 6 mg/kg of caffeine had no impact on the 20 m sprint (placebo: 3.26 ± 0.21, caffeine 3.27 ± 0.24, P > 0.05), and the 5-m shuttle run performance (placebo: 15.35 ± 0.61, caffeine: 15.56 ± 0.67, P > 0.05) (Moore et al., 2018). Moreover, a study by Romedhani et al. aimed to mitigate the adverse effects of sleep restriction on the RAST. The study found that supplementing with 5 mg/kg of caffeine after 4 h of sleep restriction increased muscle damage without improving performance compared to the placebo group (Romdhani et al., 2021). Additionally, in a study conducted by Paton et al., 16 male team athletes consumed either a placebo or 6 mg/kg of caffeine 60 min before completing a 20 m sprint anaerobic repeat sprint test involving 10 repetitions completed in 10 s each (with rest during the remainder of the 10 s). The study found no significant differences in any of the metrics between the caffeine and placebo groups (Paton et al., 2001). Running-based tests more closely resemble the activity format of a team athlete competition. Different intermittent sprint exercise patterns may affect the effects of caffeine differently. Lee et al. found that caffeine ingestion enhanced sprint performance at rest intervals of 90 s, but did not benefit repeated sprints spaced at 20-second intervals (Lee et al., 2012). This suggests that the rest intervals between sprints may modulate the enhancing effect of caffeine. In addition, a recent meta-analysis examining the impact of caffeine on RSA found that caffeine intake did not affect total power or optimal sprint performance (Lopes-Silva et al., 2019). The study suggested that the lack of a significant impact on optimal sprint performance following caffeine supplementation during repeated sprint activity may be linked to energy system utilization. Gaitanos et al. reported that during a single 6-s sprint, PCr metabolism contributed to 49.6% of total anaerobic ATP resynthesis, followed by glycolysis at 40% (Gaitanos et al., 1993). The impact of caffeine on glycolytic metabolism has yet to be demonstrated. Caffeine intake may improve intermittent exercise, which consists of short sprints lasting 10 s or less, with a recovery period of 60–300 s between sprints to allow for almost full recovery. However, caffeine does not improve RSA. During repetitive sprint exercise, there is a significant decrease in sprint performance, and as fatigue sets in, caffeine may lead to lysis due to increased anaerobic metabolism by-products. In the future, it would be beneficial further to explore the dose-effect relationship between exercise intervals and caffeine. Additionally, finding the appropriate dose of caffeine to counteract the negative effects of sleep restriction on anaerobic repetitive sprint performance could help clarify the effects of caffeine on repetitive sprint performance.

### 4.3 Limitations

This study has several limitations. The present study only included male college soccer players as participants. However, it can serve as a foundation for future studies involving women, elite athletes, and larger sample sizes. Second, the intervention strategy for sleep deprivation involved a ≤5-hour sleep restriction within 24 h. It is unclear whether the findings can be applied to longer periods of sleep restriction or deprivation, but it remains highly relevant as it reflects the most common protocol in the routines of college athletes. Third, the study utilized 3 mg/kg of caffeine as a nutritional intervention to counteract the effects of sleep restriction. It's uncertain whether the conclusions can be extended to other caffeine dosage interventions. However, 3 mg/kg is a relatively safe strategy for improving sports performance, so the results of the current study are still highly useful. Finally, the aerobic and anaerobic performance test was conducted at 9:00 a.m. The potential influence of circadian rhythms and waking hours on the effects of sleep restriction on athletic performance has not been explored. Further research is required to gain a deeper understanding of this aspect in the future.

## 5 Conclusion

Sleep restriction of ≤5 h within 24 h harms the anaerobic repeated sprint and aerobic performance in collegiate male soccer players; furthermore, acute supplementation with a 3 mg kg^−1^ dose of caffeine was effective in mitigating the negative effects of sleep restriction on aerobic endurance performance in collegiate soccer players but had no effect on anaerobic repetitive sprint performance.

Athletes should avoid sleep deprivation of ≤5 h in the 24 h before training or competition to avoid causing a decrease in sports performance on the following day. Caffeine supplementation can improve aerobic endurance under sleep restriction, but individual tolerance and potential side effects, such as anxiety or stomach discomfort, should be considered. Athletes and coaches may use caffeine to counteract sleep deprivation, but personal responses should guide its use.

## Data Availability

The raw data supporting the conclusions of this article will be made available by the authors, without undue reservation.
