# Supplementary material for: Effect of caffeine supplementation on anaerobic and aerobic performance in sleep-restricted male college soccer players
Source: Front Physiol. 2025 Mar 19;16:1561695. doi: 10.3389/fphys.2025.1561695 (PMC11961957; doi:10.3389/fphys.2025.1561695)
Supplement: Supplementary file 1 [file Table1.docx]

Supplementary Material

# Supplementary Figures and Tables

Table 1 Basic information of research participants.

| Ages (yrs) | Height (cm) | Weight (kg) | BMI (kg/m2) | Body fat rate(%) | Training years(yrs) |
| --- | --- | --- | --- | --- | --- |
| 20.0±1.3 | 174.6±6.1 | 72.2±8.2 | 23.7±2.2 | 14.9±6.1 | 10.2±1.7 |

## Supplementary Figures


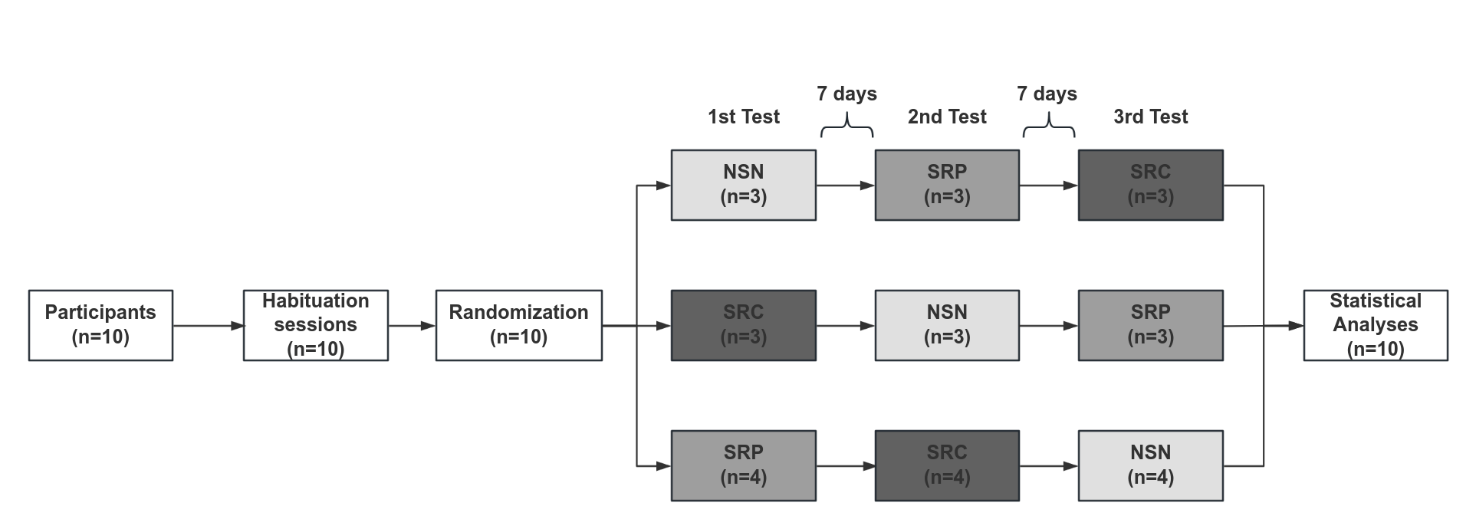


Supplementary Figure 1. Simplified experimental protocol. NSN, normal sleep night; SRP, sleep restriction supplemented with placebo; SRC, sleep restriction supplemented with caffeine; PLA indicates xylitol placebo; CAF indicates 3 mg·kg−1 of caffeine.


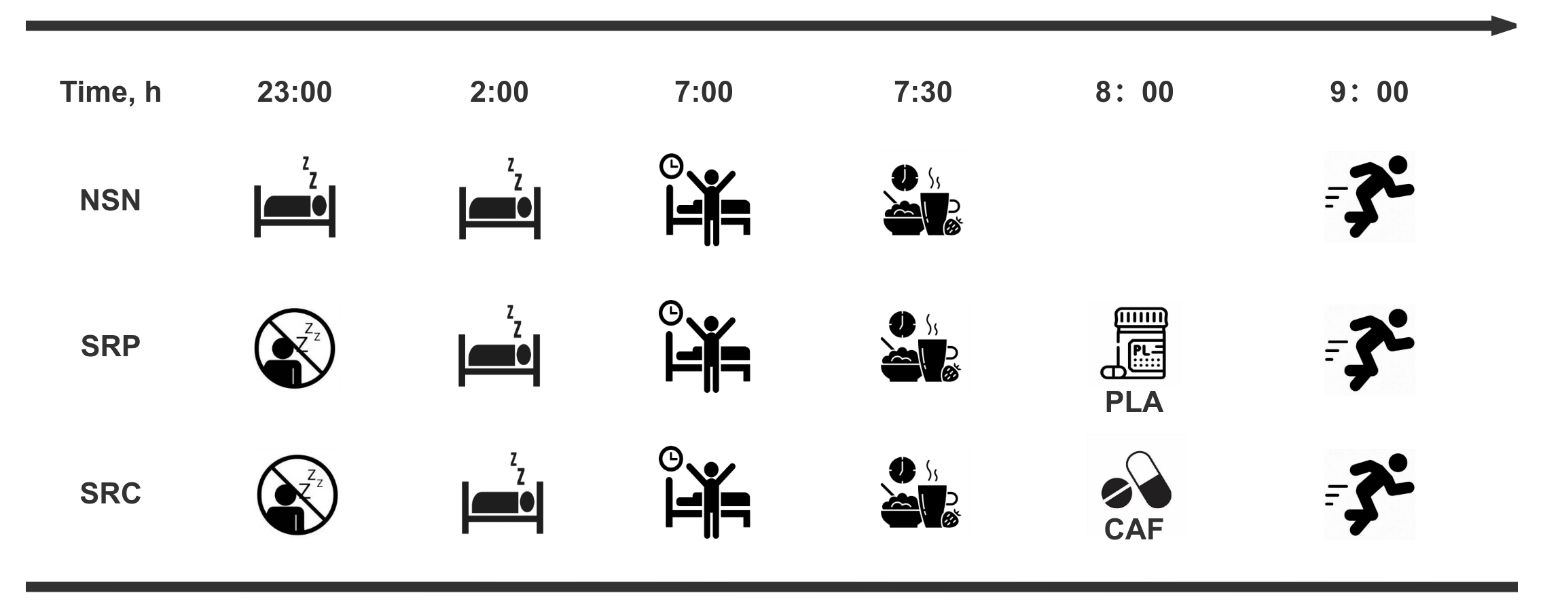


Supplementary Figure 2. Simplified sleep intervention protocol. All times are expressed in local time (GMT+8 h). NSN, normal sleep night; SRP, sleep restriction supplemented with placebo; SRC, sleep restriction supplemented with caffeine; PLA indicates xylitol placebo; CAF indicates 3 mg·kg^−1^ of caffeine.


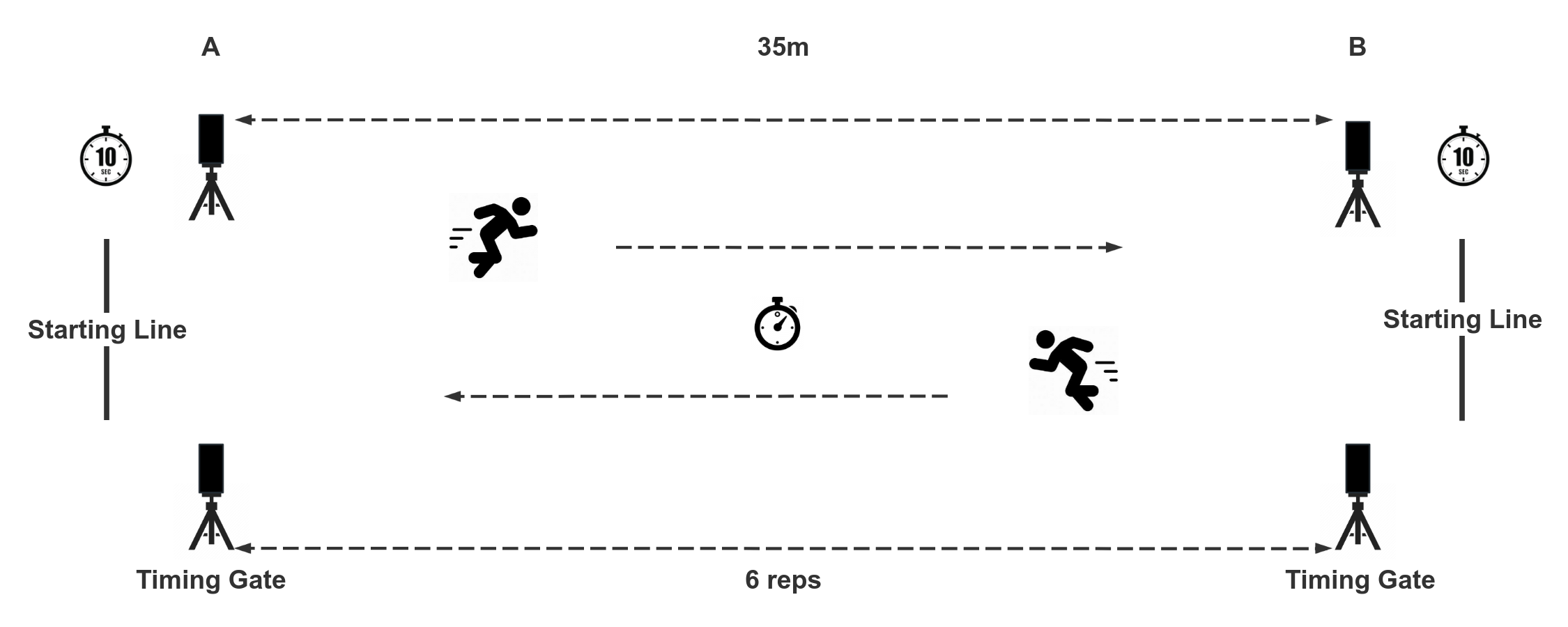


Supplementary Figure 3. Schematic representation of the Running-Based Anaerobic Sprint Test (RAST).


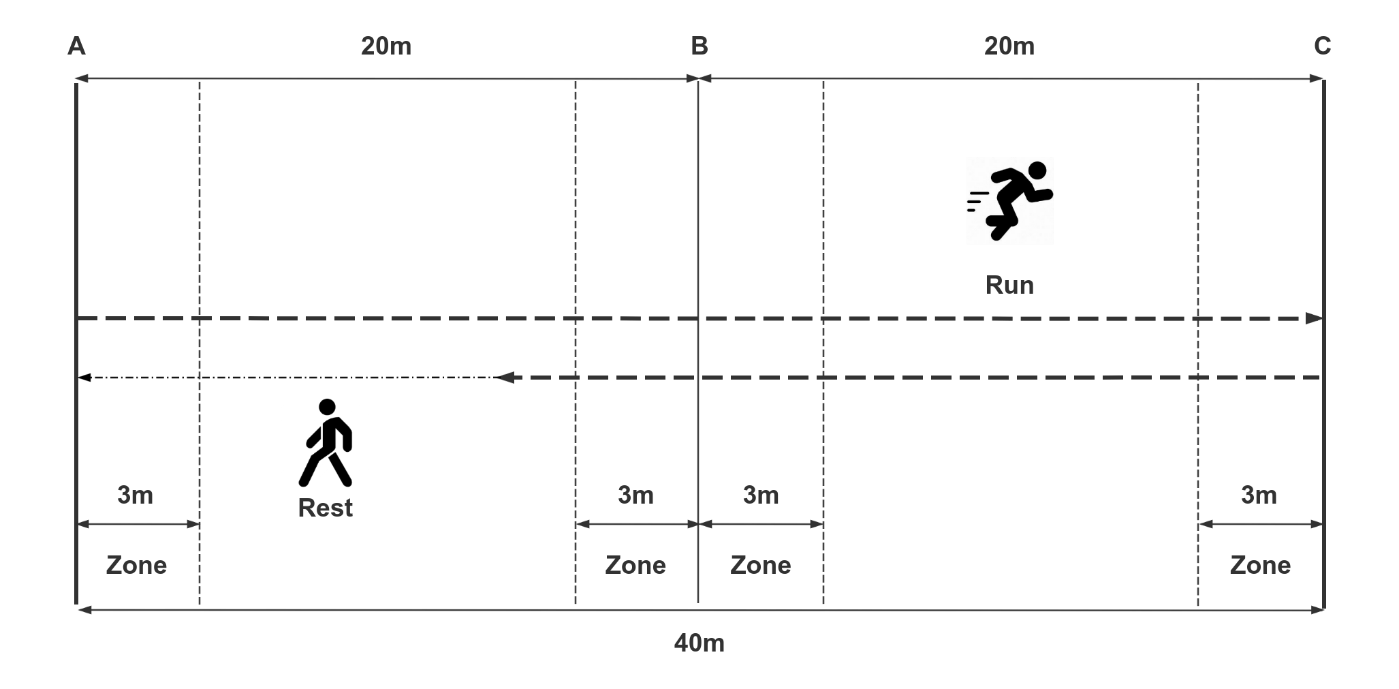


Supplementary Figure 4. Schematic representation of 30-15 Intermittent Fitness Test (30-15_IFT_).


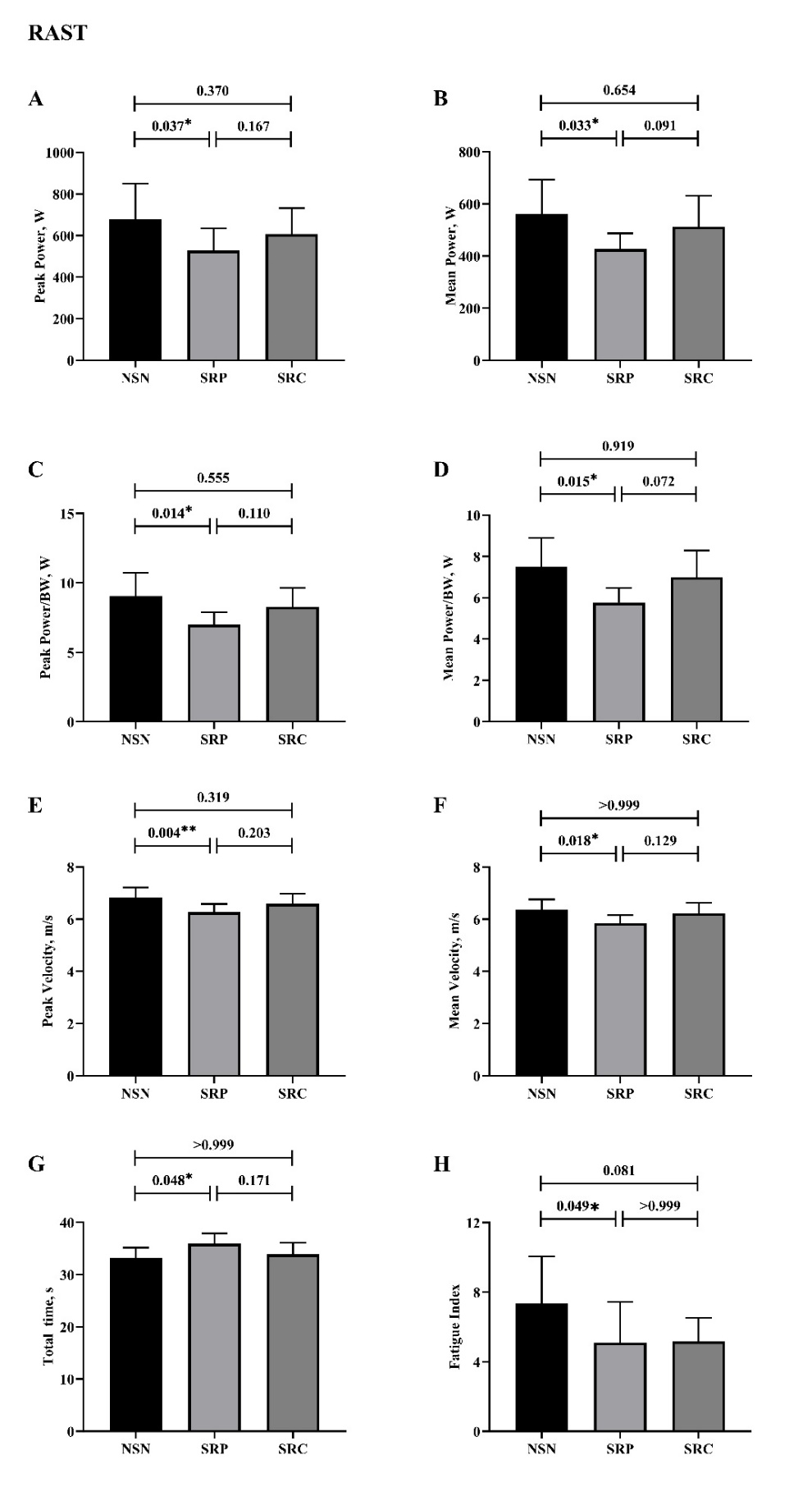


A: Results of peak power, B: Results of peak power/BW, C: Results of mean power, D: Results of mean power/BW, E: Results of peak velocity, F: Results of mean velocity, G: Results of total time, H: Results of fatigue index. * *p*<0.05, ***p*<0.01.

**Supplementary Figure 5.** Results of running-based anaerobic sprint test


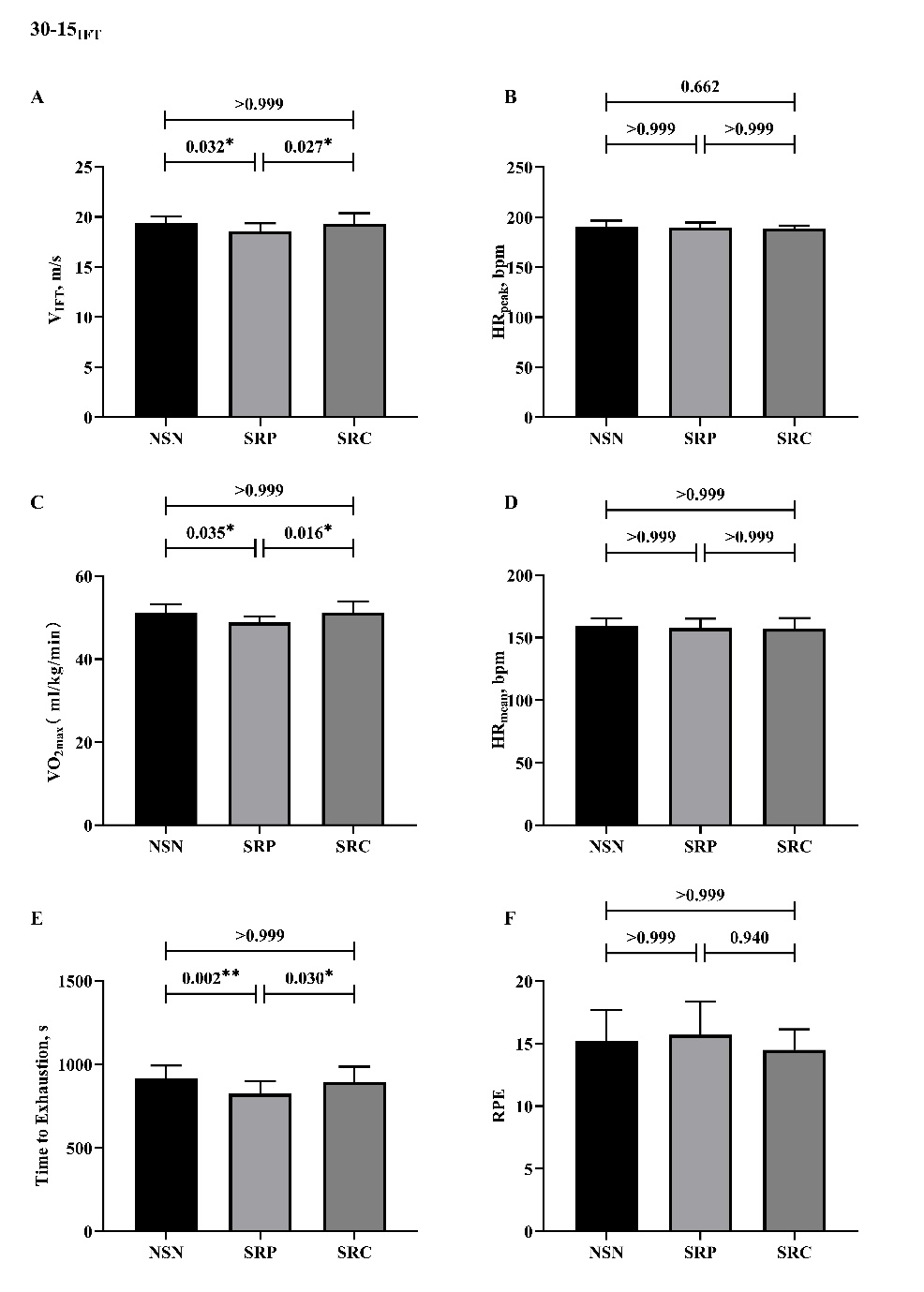


A: Results of V_IFT_, B: Results of peak heart rates, C: Results of VO_2max_, D: Results of mean heart rates, E: Results of time to exhaustion, F: Results of RPE. * *p*<0.05, ***p*<0.01.

Supplementary Figure 6. Results of 30-15 intermittent fitness test
